# Non‐Traditional Lipid Parameters and Risk of Adverse Pregnancy Outcomes in Gestational Diabetes Mellitus: Mediation by Maternal Metabolites

**DOI:** 10.1111/1753-0407.70118

**Published:** 2025-07-31

**Authors:** Jing‐yi Guo, Dan‐dan Yan, Wei Chen, Su‐na Wang, Yan‐wei Zheng, Wei‐tuo Zhang, Cheng Hu, Ming‐juan Luo, Xiang‐tian Yu

**Affiliations:** ^1^ Clinical Research Center Shanghai Sixth People's Hospital Affiliated to Shanghai Jiao Tong University School of Medicine Shanghai China; ^2^ Department of Endocrinology and Metabolism Shanghai Sixth People's Hospital Affiliated to Shanghai Jiao Tong University School of Medicine, Shanghai Diabetes Institute, Shanghai Clinical Center of Diabetes, Shanghai Key Laboratory of Diabetes Mellitus, Shanghai Key Clinical Center for Metabolic Disease Shanghai China; ^3^ Department of Gynecology and Obstetrics Shanghai Sixth People's Hospital Affiliated to Shanghai Jiao Tong University School of Medicine Shanghai China; ^4^ Clinical Research Center Shanghai Jiao Tong University School of Medicine Shanghai China; ^5^ Shanghai Diabetes Institute, Shanghai Key Laboratory of Diabetes Mellitus Shanghai Clinical Centre for Diabetes, Shanghai Sixth People's Hospital Affiliated to Shanghai Jiao Tong University School of Medicine Shanghai China; ^6^ Department of Endocrinology and Metabolism The University of Hong Kong‐Shenzhen Hospital Shenzhen China

**Keywords:** adverse pregnancy outcomes, gestational diabetes mellitus, mediation, metabolomics, non‐traditional lipid parameters

## Abstract

**Aims:**

To examine the association between non‐traditional lipid parameters and adverse pregnancy outcomes (APOs) in women with gestational diabetes mellitus (GDM) and the mediating role of maternal serum metabolites during pregnancy.

**Materials and Methods:**

This prospective observational study enrolled 399 women with GDM. Multivariate logistic regression was used to examine the association between non‐traditional lipid parameters and APOs risk. Additionally, we assessed the mediating role of single and composite maternal serum metabolites during pregnancy using causal mediation analysis and high‐dimensional mediation analysis, respectively.

**Results:**

APOs were observed in 12.0% (*N* = 48) of participants. Seven non‐traditional lipid parameters, except for the RC/HDL‐C ratio, were associated with APOs risk, with the highest estimate for the atherogenic index of plasma (AIP) (OR = 3.873, 95% CI: 1.079–13.934, *p* = 0.037) after adjusting for confounders. Maternal metabolic markers mediated these associations, with mediation effect proportions of 21.9%–39.4%. Seven key metabolic markers were identified as potential mediators primarily involved in the biosynthesis of the unsaturated fatty acids pathway. Gene set variation analysis revealed significant differences in the positive regulation of this pathway between the APO and normal pregnancy outcome groups (*p* = 0.015).

**Conclusions:**

Non‐traditional lipid parameters were positively associated with APOs risk in women with GDM. Maternal serum metabolites, predominantly involved in the biosynthesis of unsaturated fatty acids, contribute to these associations.

**Summary**

- Seven non‐traditional lipid parameters were positively associated with adverse pregnancy outcome risk in women with gestational diabetes mellitus.
- Maternal serum metabolites, predominantly involved in the biosynthesis of unsaturated fatty acids, contribute to these associations between.
- These findings provide new ideas for the etiology of adverse pregnancy outcomes in gestational diabetes mellitus, as well as new perspectives for further research into their treatment strategies.

## 1 Introduction

Gestational diabetes mellitus (GDM) is one of the most common pregnancy complications [1], affecting approximately 6%–15% of pregnant women worldwide, with its prevalence increasing in recent decades [2]. GDM poses short‐ and long‐term health risks for both mothers and their children. For mothers, GDM is a risk factor for the development of postpartum type 2 diabetes mellitus (T2DM) [3]. In offspring, GDM increases the risk of short‐term adverse pregnancy outcomes (APOs) [4, 5] and long‐term consequences, such as childhood obesity [6].

Neonatal APOs include stillbirth, neonatal death, congenital anomalies, preterm birth, macrosomia, small for gestational age (SGA) neonates, neonatal hypoglycemia, hyperbilirubinemia, respiratory distress syndrome, and low Apgar score [7, 8]. The incidence of various APO events among women diagnosed with GDM using the International Association of Diabetes and Pregnancy Study Groups (IADPSG) criteria ranges from 2.9% to 23.9%, significantly higher than that in women without GDM [9]. Advanced maternal age, pre‐pregnancy body mass index (BMI), gestational weight gain, insulin resistance, and HbA1c levels at the time of diagnosis are associated with the risk of developing APOs in women with GDM [10, 11, 12, 13]. In addition, lipid profiles, including total cholesterol (TC), triglyceride (TG), low‐density lipoprotein cholesterol (LDL‐C), and high‐density lipoprotein cholesterol (HDL‐C), are associated with APOs in women with GDM and non‐GDM [14, 15, 16], with a notably stronger association in women with GDM [17].

In recent years, non‐traditional lipid parameters, such as the lipoprotein combination index (LCI), non‐HDL‐C, residual cholesterol (RC), and atherogenic coefficient (AC), which are derived from several traditional lipid parameters, have been introduced and highlighted as complements to lipid profiles [18]. These non‐traditional lipid parameters provide more comprehensive insights than traditional lipid parameters and are intimately associated with diabetes and cardiovascular diseases [19, 20, 21]. However, the association between non‐traditional lipid parameters and the risk of APOs remains unknown, especially in women with GDM.

Recent advancements in high‐throughput metabolomic technologies have made it possible to observe changes in small‐molecule metabolite levels during pregnancy, offering a comprehensive view of the pathophysiological state of the body. These metabolites cause APOs owing to environmental influences [22, 23]. Nevertheless, few studies have attempted to integrate non‐traditional lipid parameters with metabolites to identify features with potential causal implications and provide information on the molecular pathways contributing to the progression of APOs in GDM.

Therefore, this study aimed to examine the association between LCI, RC, and other non‐traditional lipid parameters with APOs in women with GDM and the role of maternal serum metabolites during pregnancy as mediators in pregnancy using causal mediation analysis.

## 2 Materials and Methods

### 2.1 Study Participants

Participants were enrolled from pregnant women who underwent GDM screening at the University of Hong Kong Shenzhen Hospital between January 2015 and September 2018. The inclusion criteria were age > 18 years and diagnosis of GDM, which was determined using the IADPSG guideline [24]. The pregnant women participated in a 75‐g, 2‐h oral glucose tolerance test (OGTT) during 24–28 weeks of gestation. GDM diagnosis was confirmed when one or more of the venous plasma glucose levels reached or exceeded the following thresholds: fasting glucose ≥ 5.1 mmol/L, or 1‐h glucose ≥ 10.0 mmol/L, or 2‐h glucose ≥ 8.5 mmol/L. Exclusion criteria included the following: (i) major chronic diseases such as cardiovascular, cerebrovascular, hepatic, renal, and autoimmune diseases or malignant tumors; (ii) long‐term use of glucocorticoids or other medications that affect glucose and lipid metabolism; and (iii) participants refusal to provide informed consent.

The study was conducted in accordance with the principles set out in the Declaration of Helsinki and approved by the Ethics Committee of the University of Hong Kong‐Shenzhen Hospital ([2014] 98, [2017] 13). All participants signed an informed consent form.

### 2.2 Data Collection

We collected data on participants' demographic characteristics, serum lipid parameters, and neonatal pregnancy outcomes. The demographic information included age, height, pre‐pregnancy weight, pregnancy weight gain, and family history of diabetes. The BMI was calculated by dividing the mass (kg) by the square of height (m^2^). The lipid parameters, including TC, TG, HDL‐C, and LDL‐C, were measured using spectrophotometry on a Siemens ADVIA‐2400 automatic biochemical analyzer (Siemens AG, Munich, Germany). Information on neonatal pregnancy outcomes was obtained from hospital maternal and infant medical records.

### 2.3 Non‐Traditional Lipid Parameter Calculations and APOs Assessment

Eight non‐traditional lipid parameters were calculated as follows: LCI = TC × TG × LDL‐C/HDL‐C [18]; atherogenic index of plasma (AIP) = log 10(TG/HDL‐C) [25]; non‐HDL‐C = TC − HDL‐C [26]; AC = non‐HDL‐C/HDL‐C [27]; Castelli's index‐I (CRI‐I) = TC/HDL‐C [28]; Castelli's index‐II (CRI‐II) = LDL‐C/HDL‐C [28]; RC = TC − HDL‐C − LDL‐C [29]; RC/HDL‐C ratio = RC/HDL‐C [30].

In our study, APOs were defined as neonates with any of the following conditions: fetal distress, preterm birth (live birth before 37 weeks of gestation), stillbirth (fetal death at ≥ 22 weeks of gestation), SGA (body weight or height less than the 10th percentile for gestational age), macrosomia (birth weight ≥ 4000 g), hyperbilirubinemia, and neonatal malformations. Subsequently, the study participants were categorized into two groups: those with APO and those with normal pregnancy outcomes (NPO), based on the occurrence of APOs.

### 2.4 Measurement of Serum Metabolites

Serum samples from each participant were collected into centrifuge tubes and stored at −80°C until analysis. Metabolomic analysis was performed using Metabo‐Profile (Shanghai, China) [31]. Briefly, 25 μL of serum was added to a 96‐well plate and transferred to a Biomek 4000 automated workstation (Biomek 4000, Beckman Coulter Inc., California, USA). For metabolite extraction, 120 μL of ice‐cold methanol containing a portion of the internal standard was automatically added to each sample. After vortexing for 5 min, the solution was centrifuged at 4000 g for 30 min. The resulting supernatant (30 μL) was transferred to a new 96‐well plate, and 20 μL of freshly prepared derivatization reagent (3‐nitrophenylhydrazine) was added to each well. After derivatization at 30 μC for 60 min, the samples were diluted by adding 330 μL of a 50% refrigerated methanol solution and stored at −20 μC for 20 min. Further centrifugation was performed at 4000 g and 4°C for 30 min. In each well of a new 96‐well plate, approximately 135 μL of supernatant was added to mix with 10 μL of internal standard. Serially diluted derivative stock standards were added to the left well, and plates were prepared for the assay.

The chromatographic conditions were as follows: the ACQUITY UPLC BEH C18 VanGuard pre‐column (2.1 mm × 5 mm id, 1.7 μm) and ACQUITY UPLC BEH C18 analytical column (2.1 mm × 100 mm id, 1.7 μm) were used. Mobile phase A was water containing 0.1% formic acid, while mobile phase B consisted of 70% acetonitrile and 30% isopropanol; the injection volume was 5 μL, the flow rate was 0.4 mL/min, the column temperature was maintained at 40°C, and the sample manager temperature was maintained at 10°C. The gradient conditions were as follows: 0–1 min, 5% B; 1–11 min, 5%–78% B; 11–13.5 min, 78%–95% B; 13.5–14 min, 95%–100% B; 14–16 min, 100% B; 16–16.1 min, 100%–5% B; 16.1–18 min, 5% B.

The mass spectrometry parameters were as follows: the capillary voltages for the positive electrospray ionization (ESI+) mode and negative electrospray ionization (ESI−) mode were 1.5 and 2 kV, respectively; the temperature of the ion source was set at 150°C; the desolvation temperature was set at 550°C, and the desolvation gas flow rate was set at 1000 L/h. Raw data files generated by UPLC–MS/MS were processed using Masslynx software (version 4.1; Waters, Milford, MA, USA), which allows peak integration, calibration, and quantification of each metabolite.

### 2.5 Statistical Analysis

Data are presented as mean ± standard deviation (SD), median [interquartile range (IQR)], or number (percentage), as appropriate. General characteristics of the APO and NPO groups were compared using the *t*‐test or Wilcoxon rank‐sum test for continuous variables and the chi‐square test for categorical variables.

A univariate logistic regression model was used to assess the independent effects of non‐traditional lipid parameters on APOs in patients with GDM. Potential confounders, such as age, pre‐pregnancy BMI, changes in BMI during pregnancy, and family history of diabetes, were further adjusted using multivariate logistic regression.

All 200 metabolites underwent logarithmic transformation to achieve a normal distribution and were standardized for subsequent analyses. To clarify the associations between metabolites with non‐traditional lipid parameters and APOs, we conducted a multivariate linear regression with non‐traditional lipid parameters as the independent variable and metabolites as the dependent variable. Additionally, a multivariate logistic regression was conducted with metabolites as the independent variable and APOs as the dependent variable. Age, pre‐pregnancy BMI, changes in BMI during pregnancy, and family history of diabetes were adjusted as covariates in these models. Metabolites significantly associated with both non‐traditional lipid parameters and APOs underwent further analysis to assess single metabolite‐mediated effects. Metabolites with significant single‐metabolite‐mediated effects were considered key metabolic markers. To evaluate the composite mediating effects of metabolites, we performed a high‐dimensional mediation analysis using principal component analysis (PCA) of key metabolic markers and estimated the mediating effects of the first principal component (PC1). Causal mediation analyses were implemented using the R “Mediation” package, with 1000 bootstrap samples, to generate robust estimates and confidence intervals (CI).

The co‐expression analysis of the correlation networks was performed to detect metabolic communities that exhibit similar metabolic expression patterns characterized by shared key metabolic markers. These metabolic communities consisted of metabolites that had the highest 25 Pearson's correlation coefficients with common key metabolic markers. The Kyoto Encyclopedia of Genes and Genomes (KEGG) database was used to assess the metabolic function or pathway enrichment of metabolic communities, with an FDR < 0.05 considered significantly enriched. For each significant pathway, gene set variation analysis (GSVA) was performed to calculate the enrichment score of the pathways in each sample. Differences in enrichment scores between the APO and NPO groups were analyzed using *t*‐tests.

All statistical analyses were performed using R version 4.3.3. Metabolomics analyses were performed using “MetaboAnalyst 6.0” (https://www.metaboanalyst.ca/) [32], and results were considered statistically significant at a two‐tailed *p*‐value < 0.05.

## 3 Results

### 3.1 Characteristics of Participants

This study included 399 pregnant women with GDM: 48 in the APO group and 351 in the NPO group. Compared to the NPO group, the APO group had a higher average age and higher levels of TC, LCI, AIP, non‐HDL‐C, AC, CRI‐I, CRI‐II, RC, and RC/HDL‐C ratios (all *p* < 0.05). Pre‐gestational BMI, changes in BMI during pregnancy, family history of diabetes, and levels of TG, LDL‐C, HDL‐C, FPG, 1 h‐PG, and 2 h‐PG did not show any significant statistical difference between the two groups (all *p* > 0.05) (Table 1).

**TABLE 1:** Characteristics of APO and NPO groups.

Characteristics	APO group (*N* = 48)	NPO group (*N* = 351)	*p*
Age, years	30 [29, 33]	29 [28, 31]	0.023*
Pre‐gestational BMI, kg/m^2^	21.26 ± 2.71	21.05 ± 2.60	0.604
Changes in BMI, kg/m^2^	4.81 ± 1.41	4.63 ± 1.56	0.461
Family history of diabetes, *n* (%)	16 (33.3)	87 (24.8)	0.204
TC, mmol/L	6.16 [5.43, 7.52]	5.80 [5.10, 6.60]	0.038*
TG, mmol/L	2.34 [1.94, 4.06]	2.29 [1.87, 2.96]	0.076
LDL‐C, mmol/L	3.59 [2.60, 4.23]	3.21 [2.59, 3.85]	0.094
HDL‐C, mmol/L	1.90 ± 0.38	2.00 ± 0.41	0.117
LCI	27.71 [17.78, 44.71]	22.15 [14.75, 33.16]	0.007**
AIP	0.12 [−0.01, 0.34]	0.07 [−0.08, 0.22]	0.044*
Non‐HDL‐C	4.45 ± 1.16	3.93 ± 1.03	0.001**
AC	2.34 [1.76, 2.83]	1.85 [1.27, 2.50]	0.002**
CRI‐I	3.43 ± 0.81	3.05 ± 0.75	0.001**
CRI‐II	1.90 ± 0.54	1.68 ± 0.48	0.005**
RC	0.81 [0.35, 1.33]	0.54 [0.20, 0.99]	0.004**
RC/HDL‐C ratio	0.48 [0.16, 0.81]	0.28 [0.08, 0.55]	0.003**
FPG, mmol/L	4.59 ± 0.34	4.59 ± 0.36	0.906
1 h‐PG, mmol/L	9.70 ± 1.79	9.82 ± 1.38	0.612
2 h‐PG, mmol/L	8.68 ± 1.68	8.64 ± 1.43	0.863

*Note:* Continuous variables were presented as mean ± SD or median (interquartile range, IQR). Categorical variables were presented as *n* (%).

Abbreviations: 1 h‐PG, one hour postprandial glucose; 2 h‐PG, two hours postprandial glucose; AC, atherogenic coefficient; AIP, atherogenic index of plasma; APOs, adverse pregnancy outcomes; BMI, body mass index; CRI‐I, Castelli's index‐I; CRI‐II, Castelli's index‐II; FPG, fasting plasma glucose; HDL‐C, high‐density lipoprotein cholesterol; LCI, lipoprotein combine index; LDL‐C, low‐density lipoprotein cholesterol; NPO, normal pregnancy outcomes; RC, remnant cholesterol; SD, standard deviation; TC, total cholesterol; TG, triglyceride.

* *p*‐value < 0.05.

** *p*‐value < 0.01.

### 3.2 The Associations Between Non‐Traditional Lipid Parameters and the Risk of APOs

The results of both univariate and multivariate logistic regression models indicated that LCI, AIP, non‐HDL‐C, AC, CRI‐I, CRI‐II, and RC were significantly associated with APOs (all *p* < 0.05), while the RC/HDL‐C ratio was not associated with APOs (*p* > 0.05). After adjusting for age, pre‐gestational BMI, changes in BMI, and family history of diabetes, the ORs were as follows: 1.012 (95% CI: 1.003–1.023, *p* = 0.011) for LCI, 3.873 (95% CI: 1.079–13.934, *p* = 0.037) for AIP, 1.484 (95% CI: 1.128–1.950, *p* = 0.005) for non‐HDL‐C, 1.516 (95% CI: 1.172–1.971, *p* = 0.002) for AC, 1.573 (95% CI: 1.105–2.318, *p* = 0.016) for CRI‐I, 2.046 (95% CI: 1.150–3.621, *p* = 0.014) for CRI‐II, 1.633 (95% CI: 1.079–2.588, *p* = 0.027) for RC, and 1.387 (95% CI: 0.873–2.492, *p* = 0.181) for the RC/HDL‐C ratio (Table 2).

**TABLE 2:** Associations of non‐traditional lipid parameters with APOs risk of GDM.

Variables	Crude OR (95% CI)	*p*	Adjusted OR (95% CI)	*p*
LCI	1.014 (1.005–1.024)	0.004**	1.012 (1.003–1.023)	0.011*
AIP	4.722 (1.351–16.433)	0.014*	3.873 (1.079–13.934)	0.037*
Non‐HDL‐C	1.525 (1.170–1.987)	0.002**	1.484 (1.128–1.950)	0.005**
AC	1.561 (1.215–2.014)	0.001**	1.516 (1.172–1.971)	0.002**
CRI‐I	1.662 (1.178–2.425)	0.006**	1.573 (1.105–2.318)	0.016*
CRI‐II	2.197 (1.247–3.848)	0.006**	2.046 (1.150–3.621)	0.014*
RC	1.695 (1.132–2.646)	0.013*	1.633 (1.079–2.588)	0.027*
RC/HDL‐C ratio	1.484 (0.938–2.640)	0.100	1.387 (0.873–2.492)	0.181

*Note:* Models were adjusted for age, pre‐gestational BMI, changes in BMI, and family history of diabetes.

Abbreviations: AC, atherogenic coefficient; AIP, atherogenic index of plasma; APOs, adverse pregnancy outcomes; BMI, body mass index; CI, confidence interval; CRI‐I, Castelli's index‐I; CRI‐II, Castelli's index‐II; GDM, gestational diabetes mellitus; HDL‐C, high‐density lipoprotein cholesterol; LCI, lipoprotein combine index; OR, odds ratio; RC, remnant cholesterol.

* *p*‐value < 0.05.

** *p*‐value < 0.01.

### 3.3 Screening for Key Metabolic Markers

Multivariate logistic regression analysis revealed that 40 metabolites were significantly associated with APOs (all *p* < 0.05). Among these metabolites, 28 were significantly associated with LCI, 30 with AIP, 22 with non‐HDL‐C, 25 with AC, 24 with CRI‐I, 20 with CRI‐II, and 22 with RC (all *p* < 0.05) (Table S1).

Single‐metabolite causal mediation analysis was performed to screen for key metabolic markers among significant metabolites associated with both APOs and non‐traditional lipid parameters. The results indicate the following:

- Nine key metabolic markers mediated the association between LCI and APOs: l‐histidine, *cis*‐ and *trans*‐aconitic acid, isocaproic acid, citric acid, ricinoleic acid, palmitoleic acid, 10Z‐heptadecenoic acid, linoleic acid, and oleic acid (Table S2).
- Eleven key metabolic markers mediated the association between AIP and APOs: glucaric acid; *cis*‐aconitic acid; isocaproic acid; citric acid; palmitoleic acid; 10Z‐heptadecenoic acid; alpha‐linolenic acid; gamma‐linolenic acid; linoleic acid; 8,11,14‐eicosatrienoic acid; and oleic acid (Table S3).
- Seven key metabolic markers mediated the association between AC and APOs: *cis*‐ and *trans*‐aconitic acid, isocaproic acid, citric acid, palmitoleic acid, 10Z‐heptadecenoic acid, linoleic acid, and oleic acid (Table S5).
- Eleven key metabolic markers mediated the association between RC and APOs: *cis*‐ and *trans*‐aconitic acid; isocaproic acid; citric acid; ricinoleic acid; palmitoleic acid; 10Z‐heptadecenoic acid; alpha‐linolenic acid; gamma‐linolenic acid; linoleic acid; 8,11,14‐eicosatrienoic acid; and oleic acid (Table S8).
- Nine of the same key metabolic markers mediated the association of non‐HDL‐C, CRI‐I, CRI‐II, and APOs, namely, *cis*‐ and *trans*‐aconitic acid, isocaproic acid, citric acid, palmitoleic acid, 10Z‐heptadecenoic acid, alpha‐linolenic acid, gamma‐linolenic acid, linoleic acid, and oleic acid (Tables S4, S6, and S7).

### 3.4 Mediation Roles of Key Metabolic Markers in Non‐Traditional Lipid Parameters and APOs Risk Association

**FIGURE 1:**
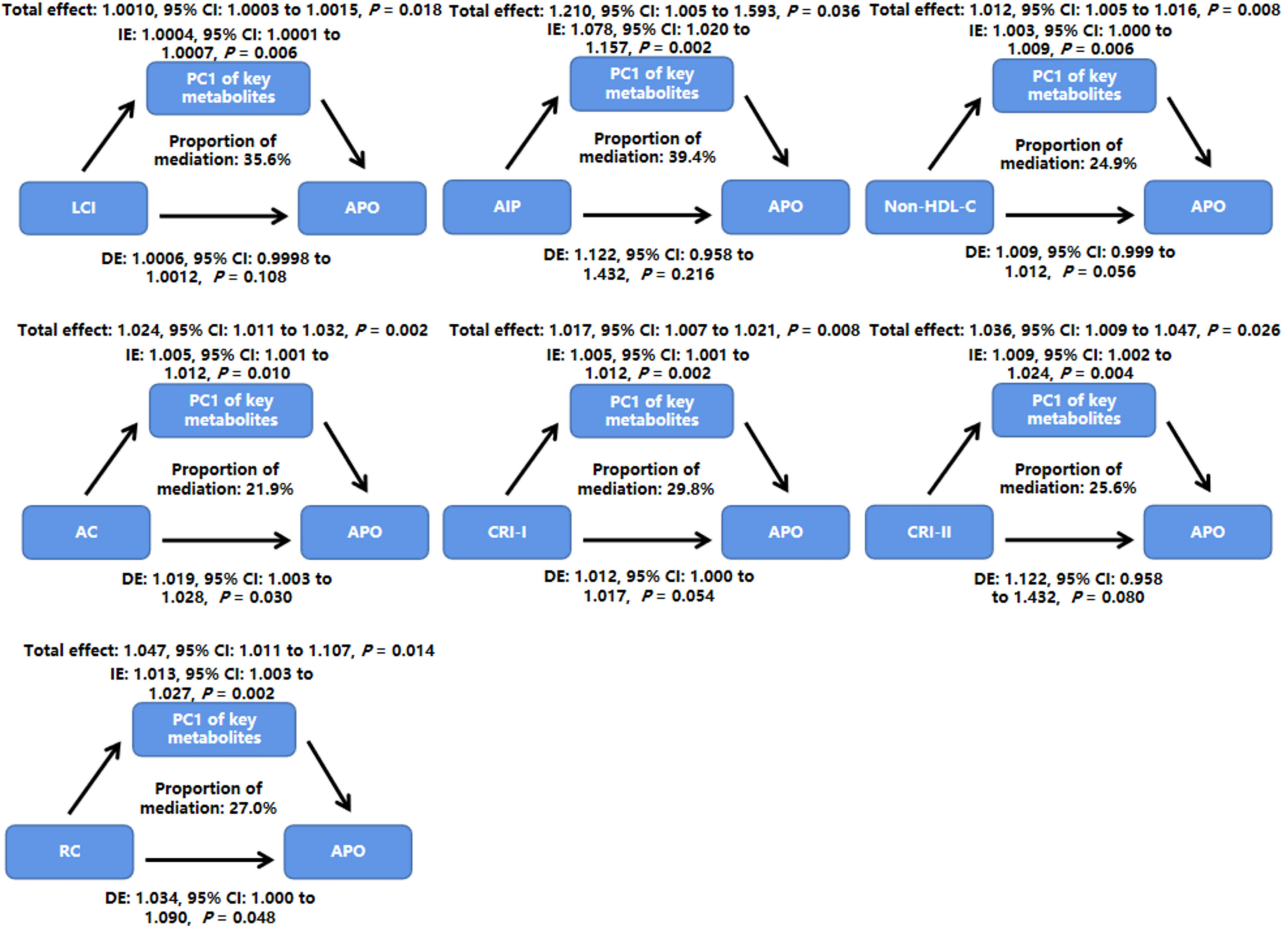
Non‐traditional lipid parameters associated with APOs are mediated by the first principal components of key metabolic markers. The models were adjusted for age, pre‐gestational BMI, changes in BMI, and family history of diabetes. Abbreviations: AC, atherogenic coefficient; AIP, atherogenic index of plasma; APOs, adverse pregnancy outcomes; BMI, body mass index; CI, confidence interval; CRI‐I, Castelli's index‐I; CRI‐II, Castelli's index‐II; DE, direct effect; HDL‐C, high‐density lipoprotein cholesterol; IE, indirect effect; LCI, lipoprotein combine index; PC1, first principal component; RC, remnant cholesterol.

The results of the causal mediation analysis indicated that PC1 of the key metabolic markers mediated the association between non‐traditional lipid parameters and APOs (all *P*‐values < 0.05). When PC1 of the key metabolic markers was used as a mediator, the proportions of mediating effects on the association of LCI, AIP, non‐HDL‐C, AC, CRI‐I, CRI‐II, and RC with APOs risk were 35.6%, 39.4%, 24.9%, 21.9%, 29.8%, 25.6%, and 27.0%, respectively (Figure 1).

### 3.5 Pathway Analysis of Metabolic Communities Related With Common Key Metabolic Markers

Seven key metabolic markers were identified as consistently mediating the role of seven non‐traditional lipid parameters associated with APOs risk. These markers include *cis* and *trans*‐aconitic acid, isocaproic acid, citric acid, palmitoleic acid, 10Z‐heptadecenoic acid, linoleic acid, and oleic acid. The co‐expression network analysis elucidated the metabolic communities of the 25 metabolites, which exhibited the highest correlation coefficients for each of the seven common key metabolic markers (Figure S1).

**FIGURE 2:**
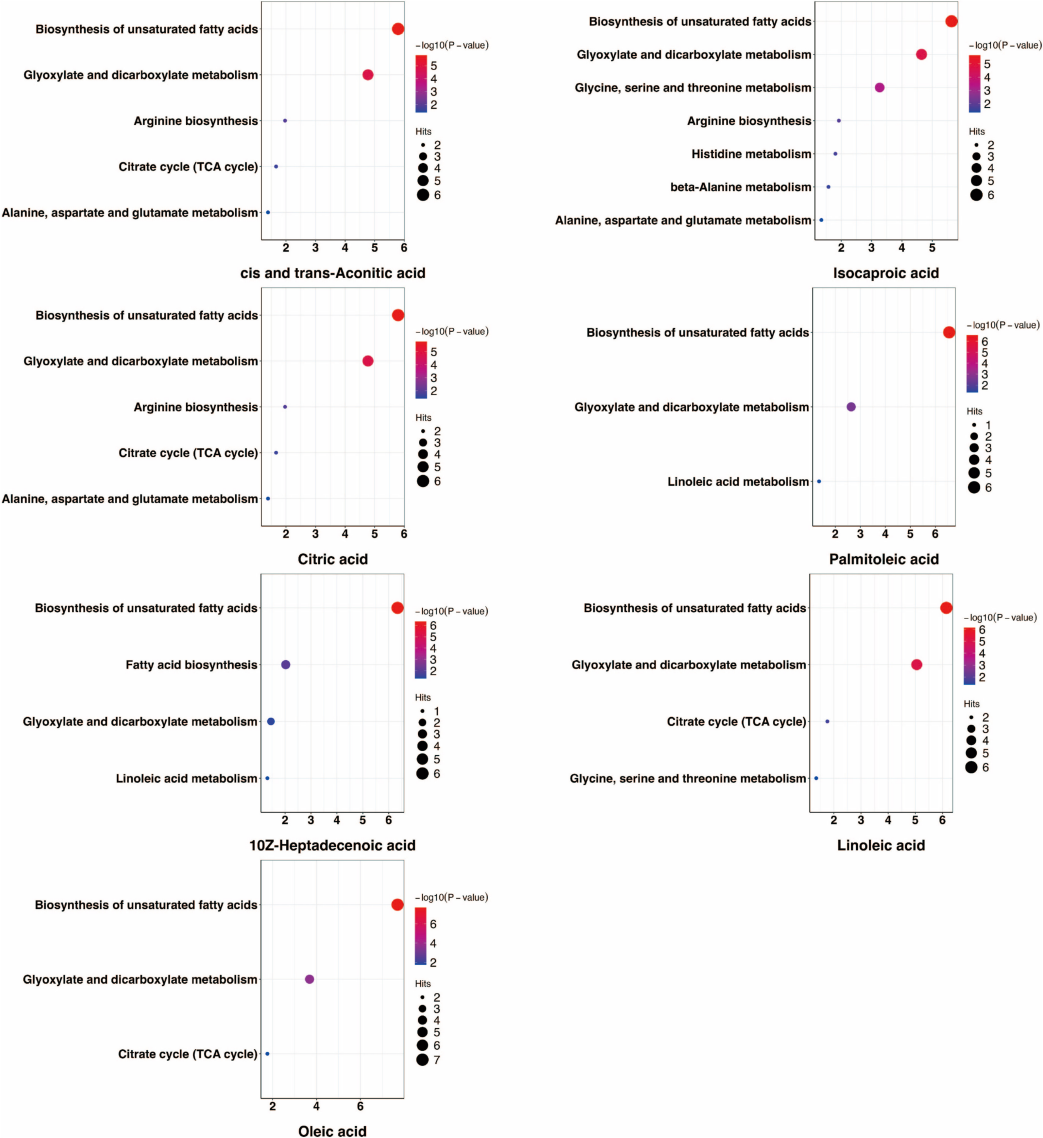
KEGG enrichment analysis of common key metabolic markers and their related metabolites in the metabolomics co‐expression network. Hits mean number of key metabolic markers in the metabolite set; *p*‐value refers to original *p‐*value in the pathway analysis.

The enriched biological functions of the metabolic communities were evaluated using the KEGG pathway enrichment analysis. The biosynthesis pathway of unsaturated fatty acid exhibited the highest fold enrichment across each metabolic community, based on seven common key metabolic markers. Glyoxylate and dicarboxylate metabolic pathways are important in metabolic communities associated with *cis*‐ and *trans*‐aconitic acid, isocaproic acid, citric acid, linoleic acid, and oleic acid. In addition, the glycine, serine, and threonine metabolic pathways were also significant in isocaproic acid‐related metabolic communities (all FDR < 0.05) (Figure 2 and Table S9).

**FIGURE 3:**
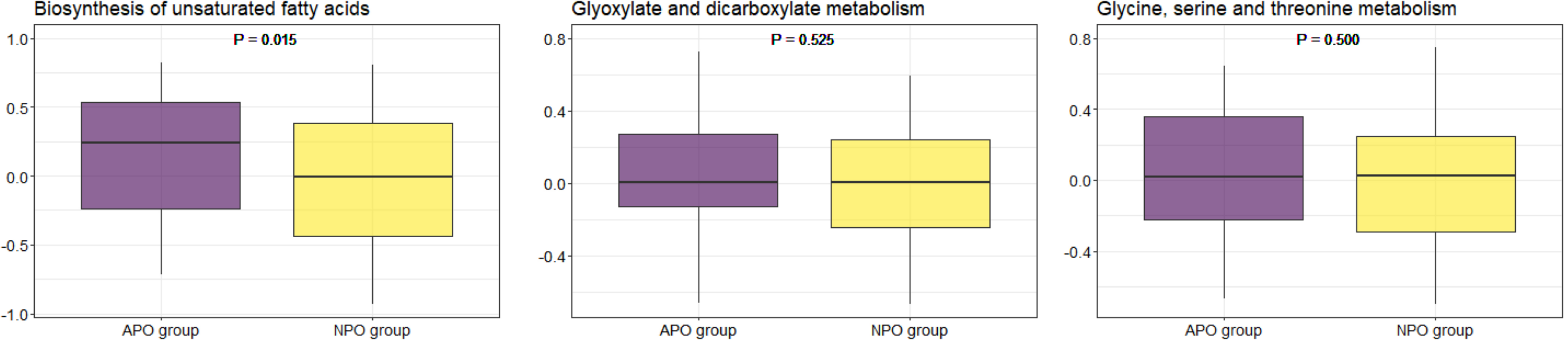
Differential enrichment scores of GSVA analysis in APO and NPO groups. Abbreviations: APO, adverse pregnancy outcomes; NPO, normal pregnancy outcomes.

The result of GSVA revealed that the biosynthesis of the unsaturated fatty acids pathway was highly activated in the APO group compared to that in the NPO group (*p* = 0.015). However, no significant differences were observed in the enrichment scores for glyoxylate and dicarboxylate metabolism, as well as glycine, serine, and threonine metabolism, between the two groups (Figure 3).

## 4 Discussion

This study examined the associations between non‐traditional lipid parameters and APO risk in women with GDM, as well as the potential mediating role of maternal metabolites in these associations. Our findings revealed that the risk of APOs in women with GDM was significantly positively associated with non‐traditional lipid parameters during pregnancy, including LCI, AIP, non‐HDL‐C, AC, CRI‐I, CRI‐II, and RC, but not with the RC/HDL‐C ratio, even after confounding factors were adjusted. In a previous study, we found that the traditional lipid parameters during pregnancy, TC (OR = 58.681, 95% CI: 1.222–2818.823, *p* = 0.039) and TG (OR = 5.900, 95% CI: 1.0881–32.210, *p* = 0.040) were significantly associated with APO risk in women with GDM, whereas HDL‐C and LDL‐C were not [14]. However, focusing only on individual lipid parameters lacks comprehensive evaluation and comparison. Non‐traditional lipid parameters are derived from multiple traditional lipid parameters, and studies suggest that their association with disease may be stronger compared to traditional lipid parameters [33, 34, 35]. Furthermore, RC, AIP, and TG/HDL cholesterol ratios have been found to be closely associated with the risk of GDM [36, 37, 38, 39, 40], even in pregnant women with normal lipid profiles [41].

Regarding the relationship between comprehensive lipid parameters and APO risk, current studies have primarily focused on TG/HDL cholesterol ratios. Recent studies have demonstrated that the maternal TG/HDL cholesterol ratio in the later stages of pregnancy is positively associated with neonatal weight and the risk of macrosomia in non‐diabetic pregnant women [42]. The second trimester TG/HDL‐C ratio has been identified as a risk factor for macrosomia and fetal distress [43], and higher TG/HDL cholesterol ratios before the 1 year of pregnancy are associated with an increased risk of APOs in general gestational women [15]. In addition, the TG/HDL‐C ratio can predict the occurrence of macrosomia in GDM, non‐GDM, and total gestational women and mediate the deleterious effect of fasting blood glucose on the risk of macrosomia [44].

We screened for key metabolic markers associated with non‐traditional lipid parameters and APOs risk using single‐metabolite causal mediator analysis. The results indicated that 14 key metabolic markers mediated the associations between the seven non‐traditional lipid parameters and APOs risk. Studies have found that the concentrations of citric, palmitoleic, oleic, linoleic, and eicosatrienoic acids are significantly higher in pregnant women with GDM compared to healthy controls [45, 46, 47, 48, 49, 50, 51]. Maternal serum levels of citric acid, 10Z‐heptadecenoic acid, ricinoleic acid, palmitoleic acid, oleic acid, linoleic acid, alpha‐linolenic acid, and gamma‐linolenic acid were significantly higher in the macrosomia group than in the control group [52]. In addition, the level of histidine in cord serum was significantly associated with macrosomia [53].

Furthermore, we assessed the composite mediating role of key metabolic markers using PCA and extracted PC1 as a potential mediator. The proportion of the mediating effect ranged between 21.9% and 39.4%. When multiple mediators are involved and exert mutual influences, analyzing each mediator separately becomes ineffective in identifying indirect effects [54]. Therefore, we used high‐dimensional mediation analysis based on PCA for the dimension reduction of multiple mediators, which is an extension of unidimensional mediation analysis [55].

To better understand the biological functions of the common key metabolic markers selected by single‐metabolite mediation analysis, we performed a co‐expression analysis of the correlation networks and further evaluated the metabolic communities using KEGG pathway enrichment. Seven common key metabolic markers were mainly involved in the biosynthesis of unsaturated fatty acids and glyoxylate and dicarboxylate metabolism pathways. Moreover, the enrichment score for GSVA in the biosynthetic unsaturated fatty acid pathway was significantly higher in the APO group (*p* = 0.015). This result is consistent with our previous findings, which analyzed the association of TG and TC with APOs risk [14]. The dysregulation of glucose and lipid metabolism, which can trigger oxidative stress through the biosynthesis of unsaturated fatty acids and glutathione metabolism, has been observed in mid‐pregnancy patients with GDM [56]. In the placentas of patients with GDM with well‐controlled glucose levels, 54 lipids and lipid‐like molecules were significantly different from those in normal controls and were significantly enriched in the biosynthesis of unsaturated fatty acids and fatty acid biosynthesis pathways [57]. Although the mechanisms involved remain unclear, some studies have suggested that cytochrome P450 is associated with the maternal inflammatory response and oxidative stress as one of the pathways by which fatty acids affect fetal development [58, 59].

Our study had several limitations. First, we performed metabolomic profiling only on serum samples from women who underwent OGTT screening in mid or late gestation; therefore, we could not assess the evolution of metabolites throughout pregnancy. Second, the biological mechanisms underlying the associations between non‐traditional lipid parameters and APOs remain unclear, although we have proposed some hypotheses regarding potential mechanisms through mediation and metabolite pathway analyses. Clinical studies based on large cohorts with dynamic monitoring of metabolites during pregnancy or animal studies are needed to validate our findings and to identify potential mechanisms.

In conclusion, our study identified seven non‐traditional lipid parameters associated with the risk of APOs in women with GDM, of which AIP had the highest estimate. These associations were mediated through maternal serum metabolites, with key metabolic markers primarily involved in the biosynthesis of unsaturated fatty acids. This is consistent with the established association of traditional lipid parameters with APOs risk. Our findings provide a new direction for investigating the pathogenesis of APOs in GDM.

## Author Contributions

C.H., M.L., and X.Y. supervised this study. J.G. and W.C. performed the data analysis. M.L. collected and checked data. X.Y., C.H., and W.Z. designed the experiments. D.Y. and Y.Z. helped carry out the experiments. J.G., D.Y., and X.Y. prepared and revised the manuscript. All authors have read and approved the final manuscript.

## Conflicts of Interest

The authors declare no conflicts of interest.

## Supporting information


**Data S1.** Supporting Information.

## Data Availability

Data will be made available on request.

## References

[1] H. D. McIntyre , P. Catalano , C. Zhang , G. Desoye , E. R. Mathiesen , and P. Damm , “Gestational Diabetes Mellitus,” Nature Reviews Disease Primers 5, no. 1 (2019): 47. 10.1038/s41572-019-0098-8 31296866

[2] X. Liu , R. Nianogo , C. Janzen , et al., “Association Between Gestational Diabetes Mellitus and Hypertension: A Systematic Review and Meta‐Analysis of Cohort Studies With a Quantitative Bias Analysis of Uncontrolled Confounding,” Hypertension 81, no. 6 (2024): 1257–1268. 38501243 10.1161/HYPERTENSIONAHA.123.22418

[3] W. Bao , E. Yeung , D. K. Tobias , et al., “Long‐Term Risk of Type 2 Diabetes Mellitus in Relation to BMI and Weight Change Among Women With a History of Gestational Diabetes Mellitus: A Prospective Cohort Study,” Diabetologia 58, no. 6 (2015): 1212–1219. 25796371 10.1007/s00125-015-3537-4 PMC4629783

[4] Y. Zhu , Q. Zheng , Y. Pan , et al., “Association Between Prepregnancy Body Mass Index or Gestational Weight Gain and Adverse Pregnancy Outcomes Among Chinese Women With Gestational Diabetes Mellitus: A Systematic Review and Meta‐Analysis,” BMJ Open 14, no. 2 (2024): e075226. 10.1136/bmjopen-2023-075226 PMC10875528 38367974

[5] D. R. Coustan , L. P. Lowe , B. E. Metzger , and A. R. Dyer , “The Hyperglycemia and Adverse Pregnancy Outcome (HAPO) Study: Paving the Way for New Diagnostic Criteria for Gestational Diabetes Mellitus,” American Journal of Obstetrics and Gynecology 202, no. 6 (2010): 654.e1–654.e6. 10.1016/j.ajog.2010.04.006 PMC2897007 20510967

[6] J.‐B. Armengaud , R. C. W. Ma , B. Siddeek , G. H. A. Visser , and U. Simeoni , “Offspring of Mothers With Hyperglycaemia in Pregnancy: The Short Term and Long‐Term Impact. What Is New?,” Diabetes Research and Clinical Practice 145 (2018): 155–166. 30092235 10.1016/j.diabres.2018.07.039

[7] Y. Wenrui , L. Cong , H. Jing , L. Chenglong , L. Zhixiong , and L. Fangkun , “Gestational Diabetes Mellitus and Adverse Pregnancy Outcomes: Systematic Review and Meta‐Analysis,” BMJ 377 (2022): e067946. 35613728 10.1136/bmj-2021-067946 PMC9131781

[8] L. Wen , Y. Chen , T. Liu , et al., “Different Subtypes of Gestational Diabetes Mellitus Are Associated With Distinct Perinatal Outcomes in Twin Pregnancies,” Diabetes Research and Clinical Practice 204 (2023): 110920. 37742804 10.1016/j.diabres.2023.110920

[9] T. P. Waters , A. R. Dyer , D. M. Scholtens , et al., “Maternal and Neonatal Morbidity for Women Who Would be Added to the Diagnosis of GDM Using IADPSG Criteria: A Secondary Analysis of the Hyperglycemia and Adverse Pregnancy Outcome Study,” Diabetes Care 39, no. 12 (2016): 2204–2210. 27634392 10.2337/dc16-1194 PMC5127228

[10] J.‐F. Ke , S. Liu , R.‐L. Ge , L. Ma , and M.‐F. Li , “Associations of Maternal Pre‐Pregnancy BMI and Gestational Weight Gain With the Risks of Adverse Pregnancy Outcomes in Chinese Women With Gestational Diabetes Mellitus,” BMC Pregnancy and Childbirth 23, no. 1 (2023): 414. 37270485 10.1186/s12884-023-05657-8 PMC10239605

[11] T. Zhang , M. Tian , P. Zhang , et al., “Risk of Adverse Pregnancy Outcomes in Pregnant Women With Gestational Diabetes Mellitus by Age: A Multicentric Cohort Study in Hebei, China,” Scientific Reports 14, no. 1 (2024): 807. 38191624 10.1038/s41598-023-49916-2 PMC10774329

[12] M. P. U. Muhuza , L. Zhang , Q. Wu , L. Qi , D. Chen , and Z. Liang , “The Association Between Maternal HbA1c and Adverse Outcomes in Gestational Diabetes,” Frontiers in Endocrinology 14 (2023): 1105899. 37008898 10.3389/fendo.2023.1105899 PMC10060951

[13] J. Lin , H. Jin , and L. Chen , “Associations Between Insulin Resistance and Adverse Pregnancy Outcomes in Women With Gestational Diabetes Mellitus: A Retrospective Study,” BMC Pregnancy and Childbirth 21, no. 1 (2021): 526. 34301212 10.1186/s12884-021-04006-x PMC8306365

[14] M. Luo , J. Guo , W. Lu , et al., “The Mediating Role of Maternal Metabolites Between Lipids and Adverse Pregnancy Outcomes of Gestational Diabetes Mellitus,” Frontiers in Medicine 9 (2022): 925602. 36035400 10.3389/fmed.2022.925602 PMC9400014

[15] N. Arbib , T. Pfeffer‐Gik , O. Sneh‐Arbib , E. Krispin , O. Rosenblat , and E. Hadar , “The Pre‐Gestational Triglycerides and High‐Density Lipoprotein Cholesterol Ratio Is Associated With Adverse Perinatal Outcomes: A Retrospective Cohort Analysis,” International Journal of Gynaecology and Obstetrics 148, no. 3 (2020): 375–380. 31811728 10.1002/ijgo.13078

[16] W.‐Y. Jin , S.‐L. Lin , R.‐L. Hou , et al., “Associations Between Maternal Lipid Profile and Pregnancy Complications and Perinatal Outcomes: A Population‐Based Study From China,” BMC Pregnancy and Childbirth 16 (2016): 60. 27000102 10.1186/s12884-016-0852-9 PMC4802610

[17] P. Shi , J. Tang , and X. Yin , “Association Between Second‐ and Third‐Trimester Maternal Lipid Profiles and Adverse Perinatal Outcomes Among Women With GDM and Non‐GDM: A Retrospective Cohort Study,” BMC Pregnancy and Childbirth 23, no. 1 (2023): 318. 37147564 10.1186/s12884-023-05630-5 PMC10161404

[18] M. Li , W. Zhang , M. Zhang , et al., “Nonlinear Relationship Between Untraditional Lipid Parameters and the Risk of Prediabetes: A Large Retrospective Study Based on Chinese Adults,” Cardiovascular Diabetology 23, no. 1 (2024): 12. 38184606 10.1186/s12933-023-02103-z PMC10771669

[19] Y. Liu , X. Jin , K. Fu , et al., “Non‐Traditional Lipid Profiles and the Risk of Stroke: A Systematic Review and Meta‐Analysis,” Nutrition, Metabolism, and Cardiovascular Diseases 33, no. 4 (2023): 698–714. 10.1016/j.numecd.2023.01.003 36737357

[20] T. Yang , Y. Liu , L. Li , et al., “Correlation Between the Triglyceride‐To‐High‐Density Lipoprotein Cholesterol Ratio and Other Unconventional Lipid Parameters With the Risk of Prediabetes and Type 2 Diabetes in Patients With Coronary Heart Disease: A RCSCD‐TCM Study in China,” Cardiovascular Diabetology 21, no. 1 (2022): 93. 35659300 10.1186/s12933-022-01531-7 PMC9166647

[21] X. H. Yang , B. L. Zhang , Y. Cheng , S. K. Fu , and H. M. Jin , “Association of Remnant Cholesterol With Risk of Cardiovascular Disease Events, Stroke, and Mortality: A Systemic Review and Meta‐Analysis,” Atherosclerosis 371 (2023): 21–31. 36966562 10.1016/j.atherosclerosis.2023.03.012

[22] K. Inoue , Q. Yan , O. A. Arah , et al., “Air Pollution and Adverse Pregnancy and Birth Outcomes: Mediation Analysis Using Metabolomic Profiles,” Current Environmental Health Reports 7, no. 3 (2020): 231–242. 32770318 10.1007/s40572-020-00284-3 PMC7599041

[23] Y. Wang , Q. Wang , L. Zhou , et al., “Metabolomics Insights Into the Prenatal Exposure Effects of Polybrominated Diphenyl Ethers on Neonatal Birth Outcomes,” Science of the Total Environment 836 (2022): 155601. 35504395 10.1016/j.scitotenv.2022.155601

[24] B. E. Metzger , S. G. Gabbe , B. Persson , et al., “International Association of Diabetes and Pregnancy Study Groups Recommendations on the Diagnosis and Classification of Hyperglycemia in Pregnancy,” Diabetes Care 33, no. 3 (2010): 676–682. 20190296 10.2337/dc09-1848 PMC2827530

[25] S. Xu , J. Liu , D. Zhao , et al., “The Association Between the AIP and Undiagnosed Diabetes in ACS Patients With Different Body Mass Indexes and LDL‐C Levels: Findings From the CCC‐ACS Project,” Cardiovascular Diabetology 23, no. 1 (2024): 77. 38378551 10.1186/s12933-024-02162-w PMC10880375

[26] S. Zelber‐Sagi , F. Salomone , H. Yeshua , et al., “Non‐High‐Density Lipoprotein Cholesterol Independently Predicts New Onset of Non‐Alcoholic Fatty Liver Disease,” Liver International 34, no. 6 (2014): e128–e135. 24118857 10.1111/liv.12318

[27] A. Otrante , A. Bounafaa , H. Berrougui , et al., “Small Dense LDL Level and LDL/HDL Distribution in Acute Coronary Syndrome Patients,” Biomedicine 11, no. 4 (2023): 1198. 10.3390/biomedicines11041198 PMC10135780 37189816

[28] A. A. Sangouni , M. Alizadeh , A. Jamalzehi , M. Hosseinzadeh , and K. Parastouei , “Garlic Supplementation Improves Intestinal Transit Time, Lipid Accumulation Product and Cardiometabolic Indices in Subjects With Metabolic Syndrome: A Randomized Controlled Trial,” Phytotherapy Research 37, no. 6 (2023): 2305–2314. 36721177 10.1002/ptr.7741

[29] B. G. Nordestgaard and A. Varbo , “Triglycerides and Cardiovascular Disease,” Lancet 384, no. 9943 (2014): 626–635. 25131982 10.1016/S0140-6736(14)61177-6

[30] G. Sheng , M. Kuang , R. Yang , Y. Zhong , S. Zhang , and Y. Zou , “Evaluation of the Value of Conventional and Unconventional Lipid Parameters for Predicting the Risk of Diabetes in a Non‐Diabetic Population,” Journal of Translational Medicine 20, no. 1 (2022): 266. 35690771 10.1186/s12967-022-03470-z PMC9188037

[31] G. Xie , L. Wang , T. Chen , et al., “A Metabolite Array Technology for Precision Medicine,” Analytical Chemistry 93, no. 14 (2021): 5709–5717. 33797874 10.1021/acs.analchem.0c04686

[32] J. Xia , N. Psychogios , N. Young , and D. S. Wishart , “MetaboAnalyst: A Web Server for Metabolomic Data Analysis and Interpretation,” Nucleic Acids Research 37, no. Web Server issue (2009): W652–W660. 19429898 10.1093/nar/gkp356 PMC2703878

[33] B. Li , X. Zhou , Y. Liu , Y. Zhang , and Y. Mu , “Remnant Cholesterol Is More Strongly Associated With Arterial Stiffness Than Traditional Lipids and Lipid Ratios in the General Chinese Population,” Journal of Atherosclerosis and Thrombosis 31, no. 5 (2024): 587–602. 38171806 10.5551/jat.64146 PMC11079499

[34] J. Ren , G. Yao , L. Ren , Y. Wang , J. Gao , and Y. Zhang , “Exploring the Associations Between Non‐Traditional Lipid Parameters and Epicardial Adipose Tissue Volume,” Angiology 76, no. 3 (2023): 33197231207264. 10.1177/00033197231207264 37843829

[35] S. Lu , M. Kuang , J. Yue , C. Hu , G. Sheng , and Y. Zou , “Utility of Traditional and Non‐Traditional Lipid Indicators in the Diagnosis of Nonalcoholic Fatty Liver Disease in a Japanese Population,” Lipids in Health and Disease 21, no. 1 (2022): 95. 36207744 10.1186/s12944-022-01712-z PMC9540727

[36] A. Zawiejska , K. Wróblewska‐Seniuk , P. Gutaj , J. Kippen , A. Gomulska , and E. Wender‐Ozegowska , “Markers of Maternal Insulin Resistance and Lipid Ratios Measured in Early Pregnancy Are Related to Adverse Fetomaternal Outcomes in Women Treated for Hyperglycemia Detected in Early Pregnancy‐Data From a Retrospective Cohort Study,” Journal of Clinical Medicine 11, no. 7 (2022): 1777. 35407384 10.3390/jcm11071777 PMC8999957

[37] I. C. R. dos Santos‐Weiss , R. R. Réa , C. M. T. Fadel‐Picheth , et al., “The Plasma Logarithm of the Triglyceride/HDL‐Cholesterol Ratio Is a Predictor of Low Risk Gestational Diabetes in Early Pregnancy,” Clinica Chimica Acta 418 (2013): 1–4. 10.1016/j.cca.2012.12.004 23262368

[38] Y. Li , X. Wang , F. Jiang , W. Chen , J. Li , and X. Chen , “Serum Lipid Levels in Relation to Clinical Outcomes in Pregnant Women With Gestational Diabetes Mellitus: An Observational Cohort Study,” Lipids in Health and Disease 20, no. 1 (2021): 125. 34587947 10.1186/s12944-021-01565-y PMC8482603

[39] W. Wang , N. Li , X. Wang , et al., “Remnant Cholesterol Is Associated With Gestational Diabetes Mellitus: A Cohort Study,” Journal of Clinical Endocrinology and Metabolism 108, no. 11 (2023): 2924–2930. 37167108 10.1210/clinem/dgad262

[40] Y. You , H. Hu , C. Cao , Y. Han , J. Tang , and W. Zhao , “Association Between the Triglyceride to High‐Density Lipoprotein Cholesterol Ratio and the Risk of Gestational Diabetes Mellitus: A Second Analysis Based on Data From a Prospective Cohort Study,” Frontiers in Endocrinology 14 (2023): 1153072. 37576966 10.3389/fendo.2023.1153072 PMC10415043

[41] Y. Gao , Y. Hu , and L. Xiang , “Remnant Cholesterol, but Not Other Cholesterol Parameters, Is Associated With Gestational Diabetes Mellitus in Pregnant Women: A Prospective Cohort Study,” Journal of Translational Medicine 21, no. 1 (2023): 531. 37544989 10.1186/s12967-023-04322-0 PMC10405385

[42] M. Yu , W. Wang , and H. Wang , “The Late‐Gestational Triglyceride to High‐Density Lipoprotein Cholesterol Ratio Is Associated With Neonatal Macrosomia in Women Without Diabetes Mellitus,” International Journal of Endocrinology 2020 (2020): 7250287. 32655633 10.1155/2020/7250287 PMC7321524

[43] C.‐Y. Yue and C.‐M. Ying , “Epidemiological Analysis of Maternal Lipid Levels During the Second Trimester in Pregnancy and the Risk of Adverse Pregnancy Outcome Adjusted by Pregnancy BMI,” Journal of Clinical Laboratory Analysis 32, no. 8 (2018): e22568. 29774596 10.1002/jcla.22568 PMC6817034

[44] C. Rao and F. Ping , “Second‐Trimester Maternal Lipid Profiles Rather Than Glucose Levels Predict the Occurrence of Neonatal Macrosomia Regardless of Glucose Tolerance Status: A Matched Cohort Study in Beijing,” Journal of Diabetes and Its Complications 35, no. 8 (2021): 107948. 34024685 10.1016/j.jdiacomp.2021.107948

[45] X. Meng , B. Zhu , Y. Liu , et al., “Unique Biomarker Characteristics in Gestational Diabetes Mellitus Identified by LC‐MS‐Based Metabolic Profiling,” Journal Diabetes Research 2021 (2021): 6689414. 10.1155/2021/6689414 PMC8211500 34212051

[46] S. Hosseinkhani , H. Dehghanbanadaki , H. Aazami , P. Pasalar , M. Asadi , and F. Razi , “Association of Circulating Omega 3, 6 and 9 Fatty Acids With Gestational Diabetes Mellitus: A Systematic Review,” BMC Endocrine Disorders 21, no. 1 (2021): 120. 34130655 10.1186/s12902-021-00783-w PMC8207652

[47] E. Ogundipe , S. Samuelson , and M. A. Crawford , “Gestational Diabetes Mellitus Prediction? A Unique Fatty Acid Profile Study,” Nutrition & Diabetes 10, no. 1 (2020): 36. 32999269 10.1038/s41387-020-00138-9 PMC7528007

[48] D. A. Enquobahrie , M. Denis , M. G. Tadesse , B. Gelaye , H. W. Ressom , and M. A. Williams , “Maternal Early Pregnancy Serum Metabolites and Risk of Gestational Diabetes Mellitus,” Journal of Clinical Endocrinology and Metabolism 100, no. 11 (2015): 4348–4356. 26406294 10.1210/jc.2015-2862 PMC4702451

[49] X. Chen , T. O. Scholl , M. Leskiw , J. Savaille , and T. P. Stein , “Differences in Maternal Circulating Fatty Acid Composition and Dietary Fat Intake in Women With Gestational Diabetes Mellitus or Mild Gestational Hyperglycemia,” Diabetes Care 33, no. 9 (2010): 2049–2054. 20805277 10.2337/dc10-0693 PMC2928361

[50] I. R. Azulay Chertok , Z. T. Haile , S. Eventov‐Friedman , N. Silanikove , and N. Argov‐Argaman , “Influence of Gestational Diabetes Mellitus on Fatty Acid Concentrations in Human Colostrum,” Nutrition 36 (2017): 17–21. 28336102 10.1016/j.nut.2016.12.001

[51] Y. Liu , Y.‐Y. Xia , T. Zhang , et al., “Complex Interactions Between Circulating Fatty Acid Levels, Desaturase Activities, and the Risk of Gestational Diabetes Mellitus: A Prospective Cohort Study,” Frontiers in Nutrition 9 (2022): 919357. 35898714 10.3389/fnut.2022.919357 PMC9313599

[52] M. Zhu , R. Sun , L. Jin , et al., “Metabolomics Profiling of Maternal and Umbilical Cord Blood in Normoglycemia Macrosomia,” Journal of Maternal‐Fetal and Neonatal Medicine 36, no. 2 (2023): 2270761. 37848386 10.1080/14767058.2023.2270761

[53] X. Xing , Y. Duan , Y. Wang , et al., “The Association Between Macrosomia and Amino Acids' Levels in Maternal and Cord Sera: A Case‐Control Study,” Nutrients 15, no. 15 (2023): 3440. 37571377 10.3390/nu15153440 PMC10421079

[54] M. G. B. Blum , L. Valeri , O. François , et al., “Challenges Raised by Mediation Analysis in a High‐Dimension Setting,” Environmental Health Perspectives 128, no. 5 (2020): 55001. 32379489 10.1289/EHP6240 PMC7263455

[55] Y. Zhao and L. Li , “Multimodal Data Integration via Mediation Analysis With High‐Dimensional Exposures and Mediators,” Human Brain Mapping 43, no. 8 (2022): 2519–2533. 35129252 10.1002/hbm.25800 PMC9057105

[56] X. Kong , Q. Zhu , Y. Dong , et al., “Analysis of Serum Fatty Acid, Amino Acid, and Organic Acid Profiles in Gestational Hypertension and Gestational Diabetes Mellitus via Targeted Metabolomics,” Frontiers in Nutrition 9 (2022): 974902. 36091252 10.3389/fnut.2022.974902 PMC9458889

[57] Y. Yang , Z. Pan , F. Guo , et al., “Placental Metabolic Profiling in Gestational Diabetes Mellitus: An Important Role of Fatty Acids,” Journal of Clinical Laboratory Analysis 35, no. 12 (2021): e24096. 34752662 10.1002/jcla.24096 PMC8649376

[58] P. C. Calder , L.‐S. Kremmyda , M. Vlachava , P. S. Noakes , and E. A. Miles , “Is There a Role for Fatty Acids in Early Life Programming of the Immune System?,” Proceedings of the Nutrition Society 69, no. 3 (2010): 373–380. 20462467 10.1017/S0029665110001552

[59] N. Umeda , T. Hirai , T. Ohto‐Nakanishi , K. J. Tsuchiya , and H. Matsuzaki , “Linoleic Acid and Linoleate Diols in Neonatal Cord Blood Influence Birth Weight,” Frontiers in Endocrinology 13 (2022): 986650. 36093109 10.3389/fendo.2022.986650 PMC9453817

